# Clinical features and gene variation analysis of COQ8B nephropathy: Report of seven cases

**DOI:** 10.3389/fped.2022.1030191

**Published:** 2023-02-09

**Authors:** Rui Liang, Xuelan Chen, Ying Zhang, Chak-Fun Law, Sijie Yu, Jia Jiao, Qin Yang, Daoqi Wu, Gaofu Zhang, Han Chen, Mo Wang, Haiping Yang, Anshuo Wang

**Affiliations:** ^1^ Department of Pediatrics, The University of Hong Kong-Shenzhen Hospital, Shenzhen, China; ^2^ Department of Nephrology, Children's Hospital of Chongqing Medical University, National Clinical Research Center for Child Health and Disorders, Ministry of Education Key Laboratory of Child Development and Disorders, Chongqing Key Laboratory of Pediatrics, Chongqing, China; ^3^ Center for Biomedicine and Innovations, Faculty of Medicine, Macau University Science and Technology, Taipa, China

**Keywords:** COQ8B, coenzyme Q10, proteinuria, chronic kidney disease, FSGS, calcinosis

## Abstract

**Objective:**

COQ8B nephropathy is a relatively rare autosomal recessive kidney disease characterized by proteinuria and a progressive deterioration of renal function, eventually leading to end-stage renal disease (ESRD). The objective is to study the characteristics and correlation between the genotype and the clinical phenotype of COQ8B nephropathy.

**Methods:**

This is a retrospective study focusing on the clinical characteristics of seven COQ8B nephropathy patients diagnosed by gene sequencing. Basic clinical information, clinical manifestations, examinations, imaging, genomes, pathology, treatments, and prognosis of the patients were reviewed.

**Results:**

Of the seven patients, two were male children and five were female children. The median age at the disease onset was 5 years and 3 months. The initial main clinical manifestations were proteinuria and renal insufficiency. Four patients had severe proteinuria, four had focal segmental glomerulosclerosis (FSGS) diagnosed by a renal biopsy, and two had nephrocalcinosis after an ultrasound was performed on them. There were no other clinical manifestations such as neuropathy, muscle atrophy, and so on in all of them. Their gene mutations were all exon variants, which were classified as heterozygous or homozygous variants by performing family verification analysis. Compound heterozygous variants were predominant in all, and all gene variants were inherited from their parents. One novel mutation, c.1465c>t, was found in this study. This gene mutation resulted from changes in the amino acid sequence, thus leading to an abnormal protein structure. Two patients with early diagnosis of COQ8B nephropathy presented with no renal insufficiency and were treated with oral coenzyme Q10 (CoQ10), and they maintained normal renal function. For the remaining five who were treated with CoQ10 following renal insufficiency, the deterioration of renal function could not be reversed, and they progressed to ESRD within a short time (median time: 7 months). A follow-up of these patients showed normal renal function with a CoQ10 supplement.

**Conclusion:**

For unexplained proteinuria, renal insufficiency, or steroid-resistant nephrotic syndrome, gene sequencing should be considered, in addition to renal biopsy, as early as possible. Timely diagnosis of COQ8B nephropathy and early supplementation of sufficient CoQ10 can help control the progression of the disease and significantly improve the prognosis.

## Introduction

Coenzyme Q10 (ubiquinone; CoQ10) is a small lipophilic molecule that participates in a series of key cellular processes. CoQ10, a cofactor of several mitochondrial dehydrogenases, shuttles electrons from complexes I and II to complex III in the mitochondrial respiratory chain. It is also one of the most important cellular antioxidants due to its redox potential (1). Mutations in several genes encoding CoQ10 (COQ2, *609825; COQ6, *614647; COQ8B, *615567; PDSS2, *610564) biosynthetic pathway enzymes are associated with the glomerular phenotype.

COQ8B (ADCK4)-associated nephropathy is related to mitochondrial dysfunction, which is caused by COQ8B gene variants. It is mainly characterized by moderate to severe proteinuria but rarely by hematuria, edema, chronic kidney disease (CKD), resistance to steroid therapy, and multiple system involvement such as neurodegeneration, dystonia, spasticity, seizures, intellectual disability, hypertrophic cardiomyopathy, retinopathy or optic atrophy, and sensorineural hearing loss as well as Crohn's disease (2–5). It is indeed one of the important causes of CKD in children. Shi et al. analyzed the etiology of 278,231 pediatric patients with CKD from 1 June 2013 to 31 May 2017, and 21.18% and 17% of glomerular diseases that were accounted for were related to congenital and genetic factors, respectively (6). Children with nephrotic syndrome, accounting for 10%–20%, were characterized by severe proteinuria, hypoproteinemia, edema, and hyperlipidemia with steroid resistance, and 20%–40% of them would gradually develop end-stage renal disease (ESRD). Sadowski et al. found a single gene in 29.5% (526 of 1,783) of families with steroid-resistant nephrotic syndrome (SRNS) that manifested before 25 years of age. Among them, 0.17% were ADCK4 (COQ8B) mutations (7). Using high-throughput DNA sequencing, Wang et al. found that ADCK4 (COQ8B) was the most common mutated gene (6.67%) in the Chinese population (120 cases), who were enrolled in multiple centers (8). Song et al. detected 20 (5.8%) patients with compound heterozygous mutations of COQ8B among patients with SRNS, non-nephrotic proteinuria, or CKD of unknown origin (3). In this study, we performed whole-exon sequencing for unexplained proteinuria, CKD, or SRNS and found seven cases of COQ8B gene mutations. We summarized the characteristics of COQ8B nephropathy through clinical manifestations, laboratory tests, gene analysis, and treatments.

## Methods

### Research object

Children with COQ8B nephropathy were diagnosed at the Children's Hospital of Chongqing Medical University and the University of Hong Kong-Shenzhen Hospital between 1 January 2013 and 30 November 2021. This study was approved by the Ethics Committee of the Children's Hospital of Chongqing Medical University (number 2018-95).

### Research methods

The basic clinical information, clinical manifestations, laboratory tests, imaging, treatments, and prognosis of the patients were collected and analyzed.

### Gene mutation detection

The peripheral blood samples of the patients were collected after obtaining informed consent from them and their families. The genomic DNA was extracted from these samples by using the Blood Genomic Column Medium Extraction Kit (Kangweishiji, China) according to the manufacturer's instructions.

Protein-coding exome enrichment was performed by using xGen Exome Research Panel v1.0 (IDT, Iowa, USA). The libraries were sequenced at an average depth of more than 100× on the high-throughput NGS platform, Illumina NovaSeq 6000 (San Diego, CA, USA), according to the manufacturer's instructions. The sequencing of raw data was filtered to remove poor-quality reads through quality control, which was then followed by the process of alignment to the reference genome (human) sequence using BWA software. After excluding the duplicated reads and performing a statistical analysis of the remaining reads, variants were called by using GATK software. The called variants were annotated on the basis of public databases for mutation records and population frequency in HGMD, ClinVar, Exome Sequencing Project, 1000 Genomes Project, and gnomAD databases. The deleterious effects of the variants were predicted *in silico*. All diagnostic variants were confirmed by using Sanger sequencing with segregation. All variants were evaluated and classified according to the American College of Medical Genetics (ACMG) clinical practice guidelines (9).

### Statistical methods

Excel application was used to analyze data. Continuous variables were described by median, and categorical variables were described by proportion.

## Result

### Basic information and clinical features

A total of 233 patients with unexplained proteinuria, CKD, or SRNS underwent whole-exome sequencing. We identified 70 (30%) pediatric patients with single-gene mutation. Seven of them who had COQ8B nephropathy were included in this study (two males and five females). The median age at disease onset was 5 years and 3 months, and the minimum age was 1 year and 1 month, and all of them were of Han nationality (Table 1). Four patients showed renal insufficiency at the beginning stage of the disease, and the remaining three had severe proteinuria. There was no malar erythema nor joint pain in them. No abnormal physical condition was identified upon an examination of the nervous system nor was there any muscle atrophy. Birth history and family history were unremarkable.

**Table 1:** Clinical characteristics of COQ8B nephropathy.

	1	2	3	4	5	6	7
Age of onset	5 years 3 months	1 year 1 month	8 years 1 month	8 years 2 months	4 years 6 months	11 years 2 months	4 years 7 months
Initial clinical manifestations	Proteinuria	Renal insufficiency	Renal insufficiency	Proteinuria	Renal insufficiency	Renal insufficiency	Proteinuria
Degree of proteinuria	Moderate	Severe	Moderate	Severe	Severe	Severe	Moderate
CoQ10	Yes	Yes	Yes	Yes	Yes	Yes	Yes
Time to deteriorate to CKD-V (month)	30	–	1	–	12	2	–
Blood purification/peritoneal dialysis	Yes	Yes	Yes	No	Yes	Yes	No
Renal transplant	Yes	Yes	–	No	Yes	Yes	–
Follow-up time	8 years	1 year	Died	2 ms	4 years	3 years 4 months	5 years

Urine protein-to-creatinine ratio: mild: 0.2–0.5 mg/mg; moderate: 0.5–2 mg/mg; severe: ≥2 mg/mg.

### Laboratory test

**Figure 1:**
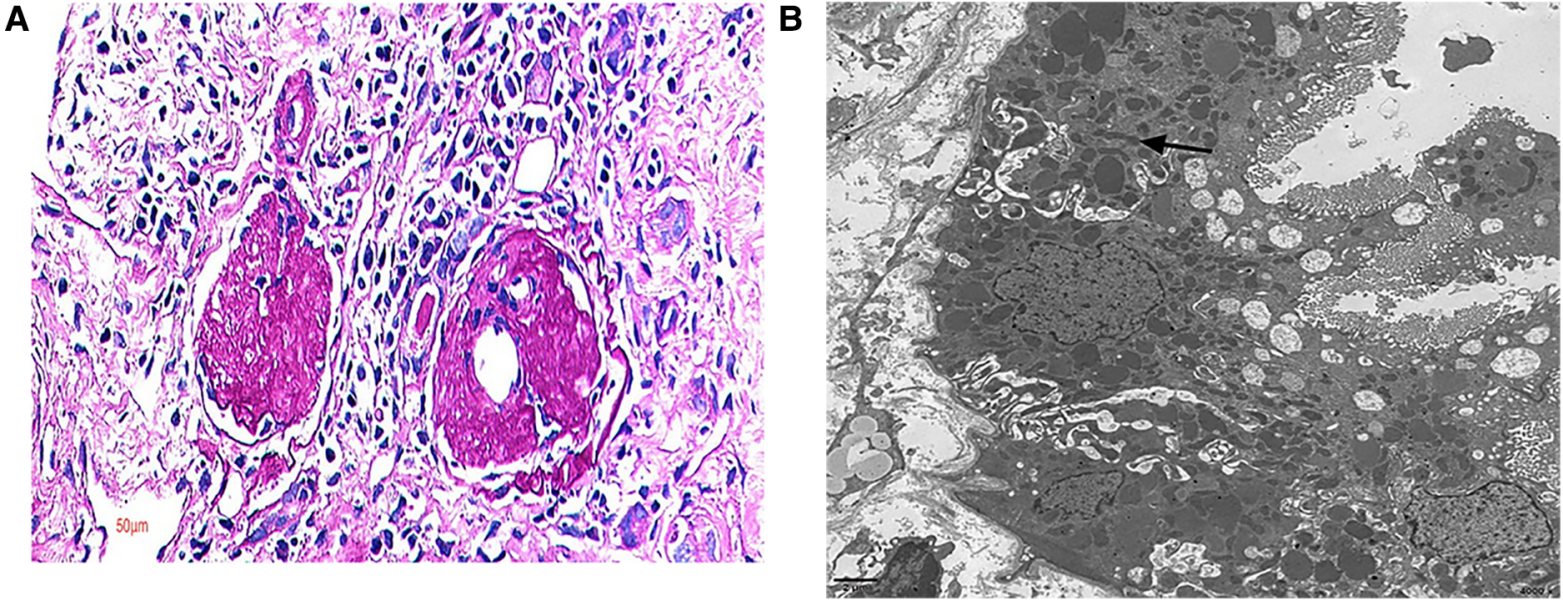
Renal pathology. Renal biopsy pathology in case 5. (**A**) Out of 18 glomeruli cases, there were 15 global sclerosis glomeruli and 1 segmental sclerosis glomerulus; light microscope, PAS 400×. (**B**) A large number of mitochondria were seen in the renal tubule, arranged crowdedly, with some of them being distorted and branched; electron microscope.

White blood cell, red blood cell, platelet, alanine aminotransferase, aspartate aminotransferase, lactate dehydrogenase, blood lipids, prothrombin time, activated partial thromboplastin time, immunoglobulin, complement, antinuclear antibody, myeloperoxidase, protease 3, and hepatitis B markers were normal (Table 2). All seven patients had no hematuria, but three had hypertension, and three presented with moderate to severe proteinuria. Four of them suffered from renal insufficiency and two had renal failure. Cases 2 and 3 showed metabolic acidosis, while echocardiography showed heart failure in case 6. Brain MR imaging in cases 2 and 3 revealed a normal condition. Eye and ear screening did not show any abnormalities in these two organs. Four patients presented with focal segmental glomerulosclerosis (FSGS) after a renal biopsy was performed on them. A large number of mitochondria were seen in the renal tubule of case 5, which were arranged crowdedly, and some of them were distorted and branched (see Figure 1). A kidney ultrasound revealed two patients with nephrocalcinosis and one with a weakened echogenicity in the renal pyramid.

**Table 2:** Laboratory examination of COQ8B nephropathy.

	1	2	3	4	5	6	7
WBC (*10^9^/L)	5.72	5.26	8.04	7.77	13.69	3.42	4.5
PLT (*10^9^/L)	238	187	215	504	351	212	230
HB (g/L)	153	95	116	148	85	98	113
NEUT# (*10^9^/L)	2.46	4.26	3.78	4.12	7.82	1.81	1.7
LYM# (*10^9^/L)	3.09	0.89	3.78	2.92	5.24	1.33	2.8
U-RBC (/uL)	3	89	1	0	2+	26.4	0
U-WBC (/uL)	1	44	2	5	0	75	0
Total protein (g/L)	52.4	43.9	47.3	59.8	53	49	51
ALB (g/L)	37.2	20	26.4	39.8	25	40.5	38
UREA (mmol/L)	5.06	35.7	18.1	5.3	22.7	3.9	6.1
CREA (umol/L)	48	234.8	185	65	772	1335.8	42.1
UA (umol/L)	490	353.3	420	373	356	556	358
K^+^ (mmol/L)	4.32	3.14	4.95	4.18	4.86	5.6	3.7
Na^+^ (mmol/L)	140.8	134.7	139	138	142	133	139
Ca (mmol/L)	2.26	1.62	2.09	2.32	1.02	1.8	2.0
Mg^2+^ (mmol/L)	0.88	0.8	0.84	0.8	1.02	0.82	0.8
Phos (mmol/L)	1.39	2.9	2.08	1.54	1.9	2.4	2.0
Total cholesterol (mmol/L)	5.45	4.26	12.06	6.01	7.52	5.04	4.3
LDH (U/L)	220	521.1	232	209	299	307	230
IgG (g/L)	5.54	4.3	3.96	7.54	3.63	9.89	5.2
IgA (g/L)	0.828	0.555	1.47	1.92	0.95	2.25	1.3
IgM (g/L)	2.98	1.17	1.88	0.963	1.02	1.6	1.61
IgE (g/L)	5.5	171	9.4	9.9	9.1	9.4	12
C3 (g/L)	0.98	0.77	0.87	1.03	0.87	0.96	0.85
C4 (g/L)	0.19	0.28	0.19	0.21	0.2	0.39	0.24
PT (s)	11	13	10.8	10.3	12.9	12.9	11.5
APTT (s)	25.6	32.8	28.4	26.8	35.8	54.8	30
Pathology	–	–	FSGS	FSGS	FSGS	–	FSGS

FSGS, focal segmental glomerulosclerosis.

### Analysis of gene sequencing

**Figure 2:**
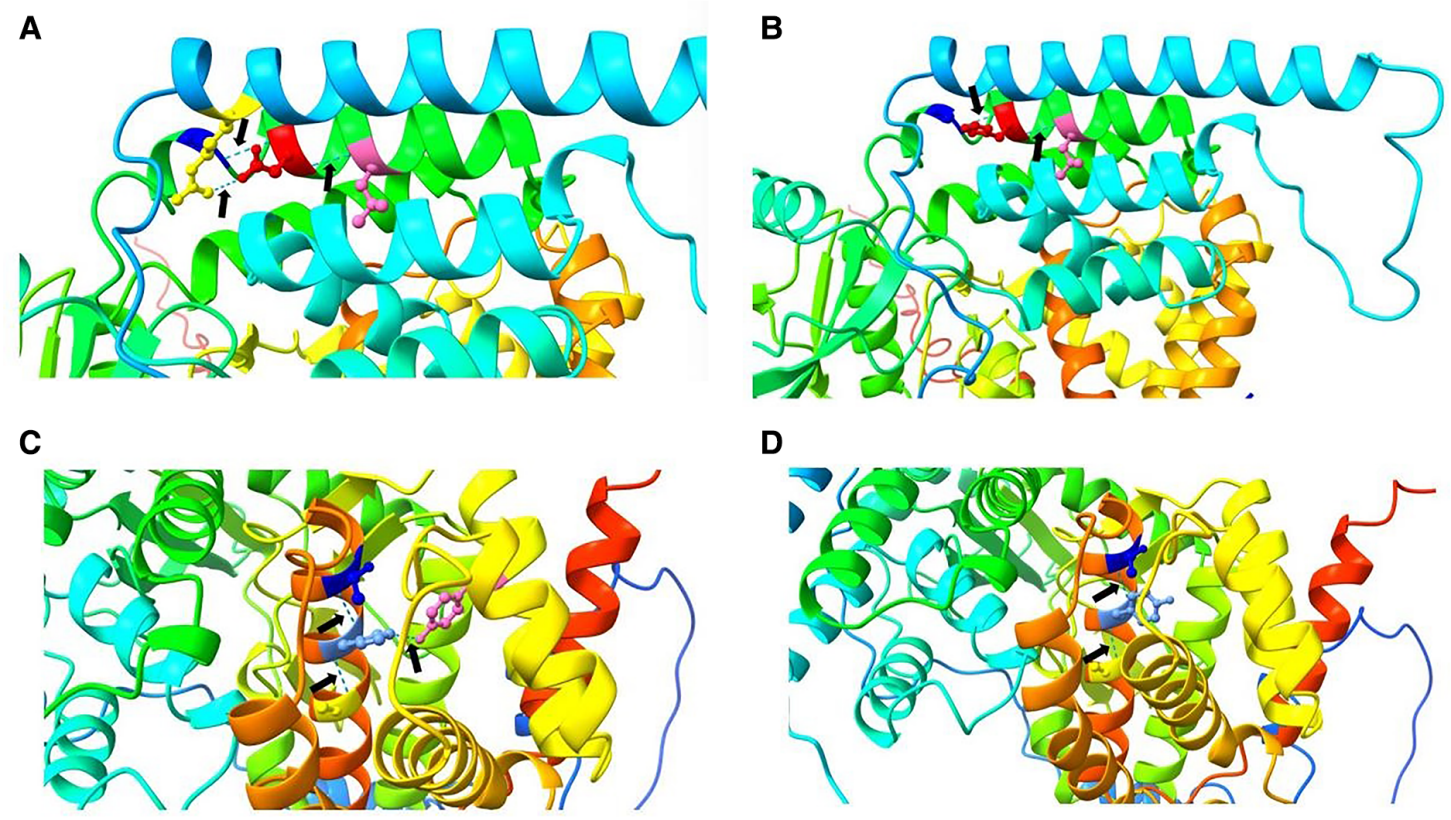
Pathogenicity analysis. (**A**) In the wild type, Asp 250 (red) forms three hydrogen bonds (black arrows) with Leu 254 (hot pink), Ser 246 (blue), and Arg 91 (yellow). (**B**) In the mutant type, 250 His (red) forms two hydrogen bonds (arrows) with Leu 254 (hot pink) and Ser 246 (blue). A change in the hydrogen bond leads to a change in the protein spatial structure, which may affect protein function. (**C**) In the wild type, His 489 (cornflower blue) forms three hydrogen bonds (black arrows) with Tyr 401 (hot pink), Thr 485 (blue), and Ala 493 (yellow). (**D**). In the mutant type, 489 Tyr (cornflower blue) forms two hydrogen bonds (arrows) with Thr 485 (blue) and Ala 493 (yellow). Again, a change in the hydrogen bond leads to a change in the protein spatial structure, which may affect protein function.

All gene mutations of the seven pediatric patients were located in the exon (Table 3), and they were analyzed by the family verification method as a heterozygous variation (5/7) or a homozygous variation (2/7). These mutations were all inherited from their parents. A total of 8 reported gene mutation sites (3 c.748g>C, 2 c.737G>A, c.625g>C, c.726_735del, c.449g>A, c.532c>t, c.472c>t, and c.1468c>t) and 1 novel mutation (c.1465c>t) were found, which involved 11 missense mutations and 1 frameshift mutation. The pathogenicity of the new site was analyzed according to ACMG guidelines (Figure 2).

**Table 3:** COQ8B genetic mutation.

	Chromosome position	Nucleic acid changes	Amino acid change	Pathogenicity analysis	Proband	Father	Mother	Mutation form
1	chr19-41209497	c.625G>C;exon8	p.D209H	Likely pathogenic	Homozygote	Heterozygote	Heterozygote	Missense mutation
2	chr19:41209497	c.748G>C;exon9	p.D250H	Likely pathogenic	Homozygote	Heterozygote	Heterozygote	Missense mutation
3	chr19-41209508	c.737G>A;exon9	p.S246N	Likely pathogenic	Heterozygote	Wild type	Heterozygote	Missense mutation
	chr19-41209509–41209519	c.726_735del;exon9	p.I243Afs*14	Likely pathogenic	Heterozygote	Heterozygote	Wild type	Frameshift mutation
4	chr19:41211271	c.449G>A;exon6	p.R150Q	Likely pathogenic	Heterozygote	Heterozygote	Wild type	Missense mutation
	chr19:41211045	c.532C>T;exon7	p.R178W	Likely pathogenic	Heterozygote	Wild type	Heterozygote	Missense mutation
5	chr19:41211248	c.472C>T;exon6	p.Q158X	Pathogenic	Heterozygote	Heterozygote	Wild type	Missense mutation
	chr19:41209497	c.748G>C;exon9	p.D250H	Likely pathogenic	Heterozygote	Wild type	Heterozygote	Missense mutation
6	chr19:41209497	c.748G>C;exon9	p.D250H	Pathogenic	Heterozygote	Heterozygote	Wild type	Missense mutation
	chr19:41198110	c.1465C>T;exon15							p.D489Y	VUS	Heterozygote	Wild type	Heterozygote	Missense mutation
7									chr19:41198107	c.1468C>T;exon15	p.R490C	Likely pathogenic	Heterozygote	Wild type	Heterozygote	Missense mutation
									chr19:41209508	c.737G>A;exon9	p.S246N	Likely pathogenic	Heterozygote	Heterozygote	Wild type	Missense mutation

### Treatment and follow-up

**Figure 3:**
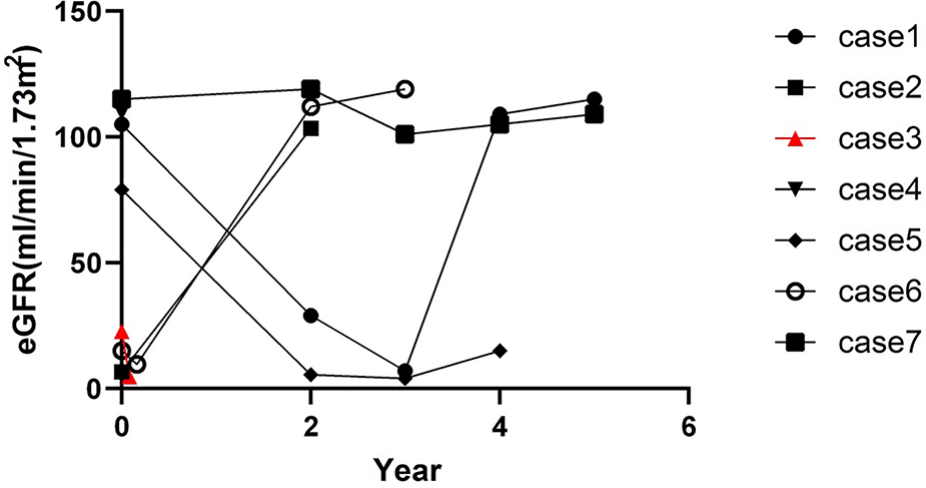
eGFR (mL/min/1.73 m^2^) follow-up. Case 1, case 2, case 5, and case 6 patients underwent kidney transplant at the 4th year, 2nd year, 4th year, and 2nd year, respectively, after disease onset. Case 3 patient died 1 month after disease onset.

All patients were treated with CoQ10 at a dosage of 5–30 mg/kg. The case 1 female patient presented with nephrotic syndrome, which was steroid-resistant. In the initial stages, neither gene testing nor a renal biopsy was done because of poor compliance. The disease progressed to CKD-V within 2 years and 6 months. The case 4 patient was treated with CoQ10 following failure of steroid treatment. However, there was no reduction in proteinuria levels. Renal function was found to be normal after a 1-month follow-up, but the follow-up period was too short to arrive at any certainty in this diagnosis. The case 7 patient was treated with CoQ10, and the renal function was found to be normal after a 5-year follow-up. Of the seven patients, there was one patient with CKD-V, two with CKD-IV, and the remaining four with renal insufficiency at the disease onset. The condition of the case 6 patient deteriorated to CKD-V after 2 months. The condition of the case 3 patient deteriorated to CKD-V after only 1 month, and unfortunately, this patient died of heart failure. The kidney function of the case 5 patient, who was diagnosed with CKD-II without a CoQ10 supplement, deteriorated 1 year after the diagnosis. These patients also underwent maintenance hemodialysis following ESRD, and the treatment procedure for one of these patients was changed to peritoneal dialysis. Four patients received renal transplantation and a continuous CoQ10 supplement, as also other immunosuppressants and antihypertensive drugs after transplantation. They were regularly followed up, following which they showed good hypertension control with no renal insufficiency nor extrarenal symptoms. The eGFR follow-up is shown in Figure 3. The median follow-up time was 3 years and 4 months.

## Discussion

COQ8B (ADCK4) is one of the genes involved in the biosynthesis of COQ10. It is located on chromosome 19q13.2 and expressed in podocytes, which are located in mitochondria inside the foot process (10). The current research cannot clearly explain the pathogenesis of COQ8B gene mutation leading to FSGS or ESRD; however, it has been found that it may be related to the reduction of podocyte migration (11). Salviati et al. found that the genotype–phenotype correlation of COQ8B mutation was not clear, and the mutation might affect the interaction between COQ8B and other COQ proteins (12). Singh et al. assumed that COQ8B (p.Asp250Asn) undermined the interaction of proteins by targeting COQ5, which is one of the important components in the COQ giant complex, resulting in a progressive deterioration from normal renal function to renal failure (13). The minimum age of such patients was 1 year and 1 month. The clinical manifestations were mainly SRNS with very few extrarenal symptoms. An ultrasound showed nephrocalcinosis in two patients. The results of all four renal biopsy cases showed FSGS. None of the seven patients had any impairment of the nervous system, heart, eyes, and ears. Schrouder et al. revealed that 98% of patients with COQ8B gene mutation had proven FSGS (14). The clinical manifestations of nephrotic syndrome, the effacement of the foot process, and a damage to the slit diagram in podocytes were observed in the COQ8B knockout animal model (15).

Kakiuchi et al. reported the first ADCK4-associated nephritis with Crohn's disease (5). Park et al. reported that in all six patients, their disease condition was accompanied by renal calcium deposition, which can be used as a diagnostic clue when combined with the effacement of foot processes and mitochondrion abnormalities in renal tubular cells (16). An ultrasound of the urinary system in case 6 indicated a weakened echogenicity in the renal pyramid, which was considered an ultrasonic manifestation after transplantation.

COQ8B nephropathy usually leads to CKD-V (2), and in this study, the condition of five children eventually progressed to ESRD. COQ8B gene mutation inhibits the migration of podocytes. However, a supplement of CoQ10 can prevent this inhibition (11). Therefore, CoQ10 should be prescribed as early as possible after diagnosis as a lifelong treatment. It is reported that oral CoQ10 can lead to proteinuria remission in such patients (11, 17–20); however, it cannot reverse the existing kidney injury. It should be emphasized that a CoQ10 continuous supplement with a dosage of 5–50 mg/kg/day can prevent the occurrence of extrarenal symptoms (2, 21).

It was reported that an empirical dosage of 15–30 mg/kg/day was an ideal treatment for COQ8B nephropathy (3). Here, the case 4 patient was treated with CoQ10 with a dosage of 30 mg/kg/day after diagnosis. However, there was no reduction in the levels of proteinuria after a follow-up of 1 month. The case 7 patient was treated with the same dosage at the early stage, and her renal function remained normal after 5 years of follow-up. As a precursor analogue of CoQ10, 2,4-dihydroxybenzoic acid is a potential treatment for these patients (1). For those patients who progressed to ESRD, renal transplantation is the optimal treatment. Song Xiaoxiang reported seven case of patients with COQ8B nephropathy without recurrence after renal transplantation (3). Four patients in this group continued to receive oral CoQ10 after renal transplantation, and their blood pressure levels and renal functions were found to be normal during follow-up.

COQ8B nephropathy is an autosomal recessive disease. A supplementation of CoQ10 before a diagnosis of renal insufficiency can reduce the progression of the disease. Therefore, early diagnosis and treatment are particularly important. The following are the recommended criteria for starting a gene examination: (1) SRNS or a significant family history of renal disease; (2) congenital (<3 months) or early-onset nephrotic syndrome (<1-year old). Some experts recommend that all individuals who exhibit SRNS before the age of 25 years undergo genetic sequencing; (3) a lack of response to immunosuppression; (4) FSGS or renal diffuse mesangial sclerosing glomerulonephritis; (5) extrarenal manifestations (syndrome); and (6) decreased glomerular filtration rate or renal failure (22). If this disease is not treated early, it could progress to ESRD within a short period of time. When this progression happens, a continuous supplementation of CoQ10 and renal replacement therapy such as hemodialysis, peritoneal dialysis, and renal transplantation are necessary. A further investigation of long-term prognosis is also necessary.

Because our study is a retrospective one, there are a few gaps in the amount of data presented. First, only a few cases of neurological complications are presented, as exemplified by the fact that we report only two cases of patients with results of cranial CT examination. Second, we show only four cases of patients with completed kidney biopsies, which could be attributed to an insufficient understanding of the risks by the patients’ families. Third and last, we could not obtain genetic information on the other family members of these patients, except that on their linear ascendants. We could also not find any related inherited family history.

## Data Availability

The data presented in the study are deposited in the GSA-Human repository, accession number HRA003933.
