# Corrigendum: Clinical features and gene variation analysis of COQ8B nephropathy: Report of seven cases

**DOI:** 10.3389/fped.2023.1183013

**Published:** 2023-03-30

**Authors:** Rui Liang, Xuelan Chen, Ying Zhang, Chak-Fun Law, Sijie Yu, Jia Jiao, Qin Yang, Daoqi Wu, Gaofu Zhang, Han Chen, Mo Wang, Haiping Yang, Anshuo Wang

**Affiliations:** ^1^ Department of Pediatrics, The University of Hong Kong-Shenzhen Hospital, Shenzhen, China; ^2^ Department of Nephrology, Children’s Hospital of Chongqing Medical University, National Clinical Research Center for Child Health and Disorders, Ministry of Education Key Laboratory of Child Development and Disorders, Chongqing Key Laboratory of Pediatrics, Chongqing, China; ^3^ Center for Biomedicine and Innovations, Faculty of Medicine, Macau University Science and Technology, Taipa, China

**Keywords:** COQ8B, coenzyme Q10, proteinuria, chronic kidney disease, FSGS, calcinosis

A Corrigendum on Clinical features and gene variation analysis of COQ8B nephropathy: Report of seven cases By Liang R, Chen X, Zhang Y, Law C-F, Yu S, Jiao J, Yang Q, Wu D, Zhang G, Chen H, Wang M, Yang H and Wang A. (2023) Front. Pediatr. 10:1030191. doi: 10.3389/fped.2022.1030191

In the published article, the accession number mentioned in the **Data Availability** statement “HRA005507” was incorrect and didn't link to the database correctly. The correct **Data Availability** statement appears below.

**Data availability statement**

The data presented in the study are deposited in the GSA-Human repository, accession number HRA003933.

The authors apologize for this error and state that this does not change the scientific conclusions of the article in any way. The original article has been updated.

